# Reverse total shoulder arthroplasty for failed treatment of proximal humerus fractures

**DOI:** 10.5152/j.aott.2021.20387

**Published:** 2021-11-01

**Authors:** Gökhan Karademir, Onur Tunalı, Ali Erşen, Sercan Akpınar, Ata Can Atalar

**Affiliations:** 1 Department of Orthopedics and Traumatology, Acıbadem Maslak Hospital, İstanbul, Turkey; 2 Department of Orthopedics and Traumatology, Acıbadem Mehmet Ali Aydınlar University, School of Medicine, İstanbul, Turkey; 3 Department of Orthopedics and Traumatology, İstanbul University, İstanbul School of Medicine, İstanbul, Turkey; 4 Department of Orthopedics and Traumatology, Ortogrup Clinic, Adana, Turkey

**Keywords:** Reverse total shoulder arthroplasty, Failed proximal humerus fractures, Fracture sequelae, Clinical and radiological assessment

## Abstract

**Objective:**

The aim of this study was to evaluate the functional and radiological outcomes and complications of reverse total shoulder arthroplasty (RTSA) for failed treatment of proximal humerus fractures (FTPHF).

**Methods:**

In this retrospective study, 20 patients (17 female, 3 male; mean age = 71.35 years; age range = 54-81 years) who underwent RTSA for FTPHF between 2012 and 2018 were included. The mean follow-up was 37.85 (range: 24-83) months. Outcome measures included shoulder range of motion, Constant score, ASES (American Shoulder and Elbow Surgeons) score, visual analog scale (VAS). Intra-or post-operative complications were also recorded.

**Results:**

The mean anterior flexion and external rotation improved from 37.25°±10.59° and 11.05°±4.79° preoperatively to 105.53° ± 9.33 and 22.37° ± 4.12° postoperatively, respectively (*P* < 0.01 for both). The mean Constant and ASES scores ameliorated from 21.95 ± 3.57 and 18.15 ± 4.69 preoperatively to 61.7 ± 7.6 and 71.18 ± 4.69 at the final follow-up, respectively (*P* < 0.01 for both). VAS significantly reduced from 6.83 ± 2.04 preoperatively to 1.79 ± 0.61 at the final follow-up (*P* < 0.01). None of the patients had major complications or required revision.

**Conclusion:**

Treatment with the RTSA for the FTPHF seems to be an effective treatment method that can provide satisfactory radiological and functional outcomes with low complication rates.

**Level of Evidence:**

Level IV, Therapeutic Study

## Introduction

Proximal humerus fractures account for 5% of all fractures in adults and 10% of all fractures in individuals aged >65 years.^1,2^ The incidence of these fractures has been increasing, especially among the elderly population.^3^ Low bone density, which is often concomitant in this age group, has been described as one of the challenges encountered in the treatment of these fractures.^3^ It has been reported that 80% of proximal humerus fractures are conservatively treated, whereas 20% require surgical treatment.^4^ The primary surgical treatment options include open reduction with internal fixation (ORIF), hemiarthroplasty, and reverse total shoulder arthroplasty (RTSA).^3,5^ After both conservative and surgical treatments, failure may be observed because of tuberosity malunion or nonunion, avascular necrosis, joint stiffness, infection, or instability.^5-^^7^ In such cases, patients may experience considerable pain and very limited functionality; moreover, their quality of life may decrease. The failed treatment of proximal humerus fractures (FTPHF) has been reported as one of the most challenging conditions in shoulder reconstruction.^6,8^

The success of the treatment of the FTPHF with the RTSA has been evaluated in a limited number of studies. Besides, reported clinical outcomes and complication rates are controversial.^5,6,8,9^ Although it has been seen as a salvage procedure with limited benefit for a long time,^6,9^ recent studies have reported that it can be an effective treatment modality for the FTPHF.^10–12^ Therefore, the present study aimed to investigate the radiological and functional outcomes as well as the complications of treatment with the RTSA for the FTPHF. Our hypothesis was that the RTSA would provide favorable functional outcomes and significantly reduce pain, especially with low complication rates unlike what was previously reported.

## Materials and Methods

**Figure 1.**
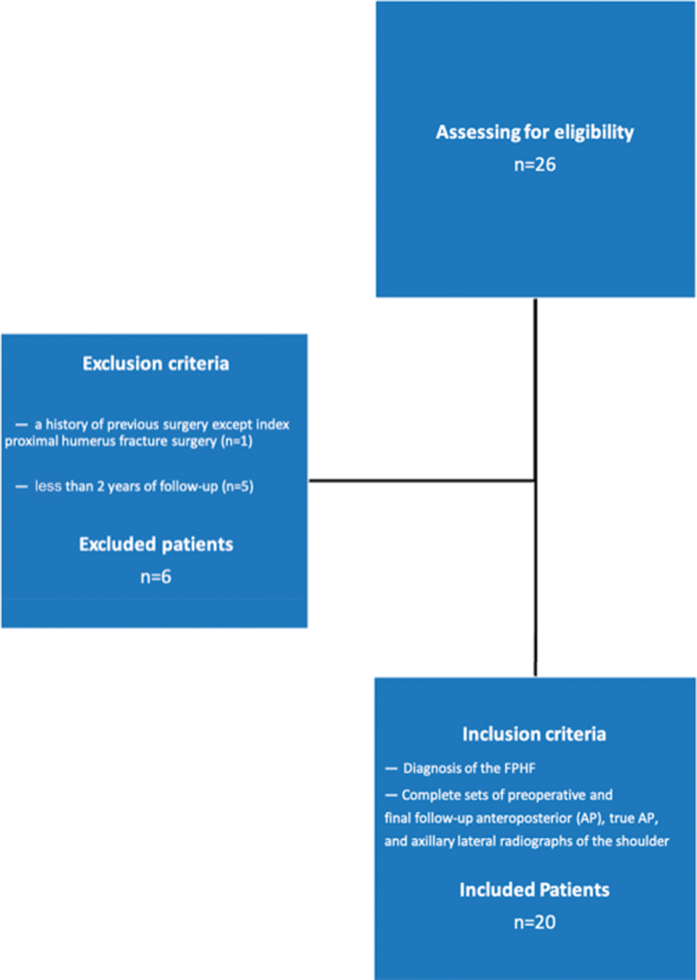
Flow diagram of the study participants.

The study was approved by our institutional review board (no: 20.120.829-16.03.2020). The clinical data of 20 patients treated with the RTSA for the FTPHF by two surgeons, who had experience over 20 years on shoulder and elbow surgery, at two different institutions between October 2012 and October 2018 were retrospectively reviewed. Inclusion criteria for the study were the following: a diagnosis of the FTPHF; complete sets of preoperative (the last pre-RTSA radiograph) and final follow-up anteroposterior (AP), true AP, and axillary lateral radiographs of the shoulder. Exclusion criterion was a history of previous surgery, except index proximal humerus fracture surgery, lower than 2 years of follow-up (Figure 1). The mean age of the patients at the time of the surgery was 71.35 (range: 54-81) years; 17 patients were females and 3 were males. The mean time between index treatment and the RTSA was 21.25 (range: 1-86) months. The mean follow-up duration after the RTSA was 37.85 (range: 24-83) months (Table 1). The indications for the surgery were pain and functional limitation. The patients were evaluated in terms of functional scores, pain scores, range of motion, time between fracture and treatment with the RTSA, and complications (Tables 2 and 3).

Preoperative and postoperative functional outcomes were evaluated using the American Shoulder and Elbow Surgeons (ASES) score and Constant score, whereas pain was evaluated using visual analog scale (VAS) score. Shoulder AP, true AP, and axillary lateral radiographs were routinely performed during radiological evaluations. Computed tomography was performed for each patient during preoperative planning to determine bone stock and evaluate tubercles and deformity.

**Figure 2. a-d.**
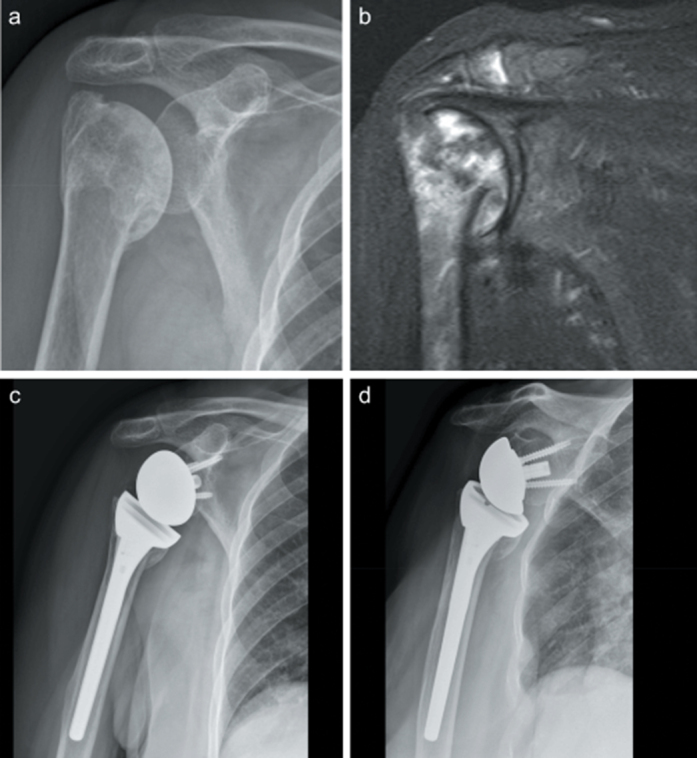
A 71-year-old woman falls onto her right shoulder sustaining proximal humerus fracture. The patient who was treated conservatively presented with persistent severe right shoulder pain and functional impairment 5 months after the injury. The patient who had varus deformity (a) and avascular necrosis of the humeral head (b) was treated with the RTSA (c, d).

**Figure 3. a, b.**
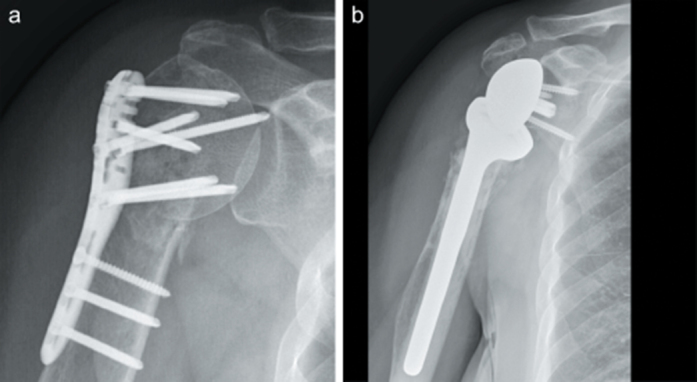
A 66-year-old male patient sustains shoulder pain that comes along with a crepitation and grinding sensation 1-month after ORIF with a locking plate for proximal humerus fracture. X-ray graphs reveal loss of reduction and screw penetration (a). The patient who has rheumatoid arthritis and osteoporosis was treated with a cemented RTSA after 1 year from index surgery (b).

**Figure 4. a, b.**
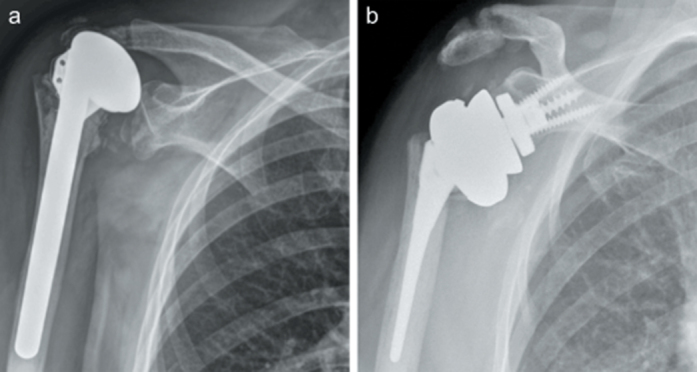
A postoperative 26th-month x-ray of a 65-year-old female patient treated with hemiarthroplasty for proximal humerus fracture reveals superior migration, acetabularization of the acromion, and glenoid erosion (a). RTSA using the cement-in-cement technique was performed after failed hemiarthroplasty (b).

Overall, five patients who had been treated conservatively (Figure 2), nine patients who had undergone ORIF using plate and screws (Figure 3), and six patients who had undergone hemiarthroplasty (Figure 4) were included in this study. The surgeries were performed by two surgeons who were experienced over 20 years in shoulder surgery. All the surgeries were performed in the beach chair position. The deltoid split approach was preferred in 1 patient and the deltopectoral approach in 19 patients. Twelve patients underwent subscapularis repair, and one patient underwent surgery without lifting the subscapularis. If present, supraspinatus and bicep tendons were resected in the patients and infraspinatus tendons were preserved if it was possible. Tubercles were not present in four patients. Malunion required tubercle osteotomy in four patients, whereas tubercle osteotomy was not performed in 12 patients. *Depuy Delta Extend* (DePuy Synthes, Warsaw, IN) brand shoulder prostheses were used in 10 patients, and *Biomet* (Biomet Inc, Warsaw, IN) brand prostheses were used in 10. Cement was used in 11 patients. Three patients were operated three times before treatment with the RTSA. One of these patients had been treated with ORIF and revised with hemiarthroplasty after failure. An antibiotic spacer was placed in the third surgery due to infection, which occurred following hemiarthroplasty. The second patient had been treated with ORIF initially. He underwent implant removal and debridement due to infection in the second operation, and an antibiotic spacer was placed in the third operation. The third patient had been treated with ORIF initially, and he had undergone revision due to nonunion. He was treated with grephonage and ORIF in the third operation in our clinic. Except for the five patients who were treated conservatively, 12 patients had undergone one previous surgery for proximal humerus fracture. Patients were subjected to routine clinical and radiological evaluations at 2 weeks, 6 weeks, 3 months, and 6 months postoperatively. Postoperative evaluations were based on the data obtained at the last follow-up examination. In terms of comorbidities, five patients had hypertension, three had hypothyroidism, two had rheumatoid arthritis, two had diabetes mellitus, two had coronary artery diseases, and one had Parkinson’s disease.

## Statistical analysis

The Wilcoxon signed-rank test was used to compare preoperative and postoperative data, and SPSS (Statistical Package for Social Sciences for Mac ver.20, IBM Corp., Armonk, NY, USA) was used for statistical analysis. *P* value of < 0.05 was considered statistically significant.

## Results

**Figure 5.**
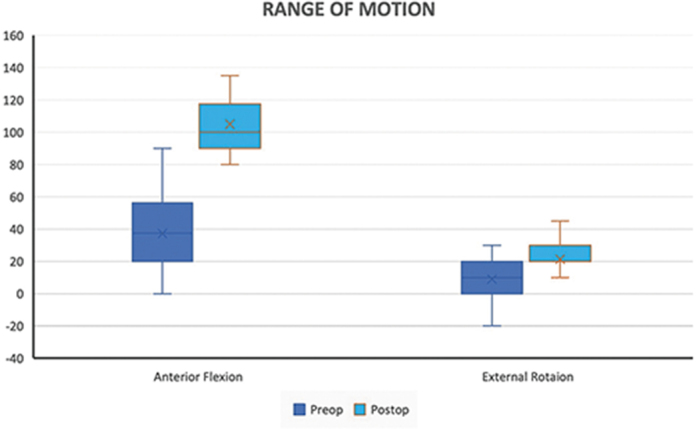
Anterior flexion and external rotation values were compared by the preoperative to the postoperative values.

**Figure 6.**
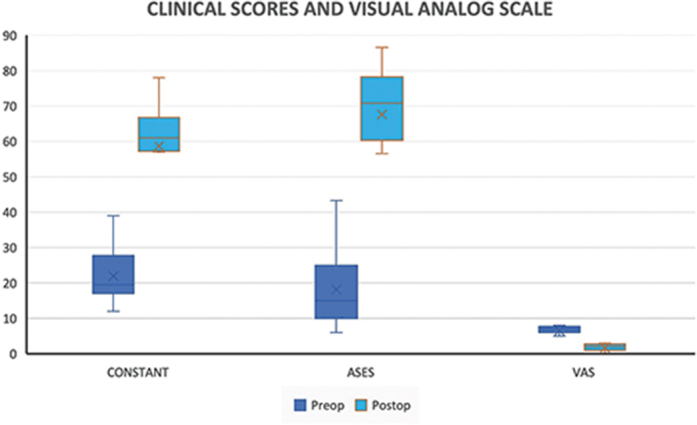
Constant score, ASES (American Shoulder and Elbow Surgeons) score, VAS (visual analog scale) score values were compared by the preoperative to the postoperative values.

The preoperative anterior flexion of the patients was 37.25° ± 10.59°and external rotation was 11.05° ± 4.79°, postoperative anterior flexion was 105.53° ± 9.33°and external rotation was 22.37° ± 4.12°(*P* < 0.001, *P* = 0.003; respectively) (Table 3) (Figure 5). While preoperative Constant and ASES scores were 21.95 ± 3.57 and 18.15 ± 4.69, respectively; they were 61.7 ± 7.6 and 71.18 ± 4.69, respectively, at the last follow-up (*P* < 0.01, *P* < 0.01; respectively) (Table 3) (Figure 6). The VAS scores of the patients decreased from a mean preoperative VAS score of 6.83 ± 2.04 to 1.79 ± 0.61 at the last follow-up (*P* < 0.01) (Table 3) (Figure 6).

Radiological evaluation revealed no loosening of prosthetic components in any patient. In four of the patients who underwent tubercle osteotomy, union was achieved in the tubercles. Grade 1 scapular notching was detected in four patients, and grade 2 scapular notching was detected in one patient according to the Sirveaux classification. Favorable functional outcomes could not be obtained in one patient because Parkinson’s disease was present as the comorbidity. However, the patient had significant pain relief following the surgical treatment. None of the patients reported major complications or required revision (Table 2).

## Discussion

The RTSA has been considered as a salvage procedure for the FTPHF with limited recovery and high complication rates for a long time.^6-^^9,13–15^ Although the outcomes and complication rates of the RTSA treatment may vary depending on the index treatment of the FTPHF, it is noticeable that better outcomes and relatively lower complication rates have been reported in recent studies, while in the past high complication rates used to be reported.^10–12,16^

Especially if the index treatment is conservative, notable improvement in functional scores can be achieved with low complication rates in patients treated with the RTSA.^10,17^ In our study, the Constant score of five patients, whose index treatment was conservative, increased from 26.2 ± 6.4 to 67 ± 12.7 (*P* < 0.01). In the series of Santana et al.,^10^ nine of 27 patients were treated with the RTSA for the FTPHF after conservative treatment, and 18 patients were treated with the RTSA for the FTPHF following the ORIF.^10^ Although successful functional outcomes were reported for both groups (*P* < 0.01), it was emphasized that treatment with the RTSA provided better outcomes in the group whose index treatment was conservative compared with the group whose index treatment was ORIF (*P* = 0.02).^10^ The largest study in which the RTSA was applied for failed ORIF of proximal humerus fracture was conducted by Grubhofer et al.^16^ It was reported that the Constant score increased from 26 (range: 4-54) to 55 (range: 19-80) in their series of 44 patients. In parallel to Grubhofer et al., the mean Constant score increased from 21.3 ± 8.6 to 54.3 ± 18.3 for this subset in our study. Levy et al. stated that the ASES score increased from 22.3 (range: 0-57.5) to 52.1 (range: 0-92.5), with the RTSA in their series consisting of 29 patients who were initially treated with hemiarthroplasty.^6^ Holschen et al. reported that the mean ASES score of 35 patients whose hemiarthroplasties were converted to the RTSA increased from 19.2 ± 3.6 to 58.5 ± 19.4.^12^ In our study, the mean preoperative ASES score of six patients whose index treatment was hemiarthroplasty was 12.4 ± 6.7. The mean postoperative ASES score of these patients increased to 74 ± 9.8 (*P* < 0.01) with the RTSA treatment.

Although favorable functional scores are commonly reported in the RTSA treatment of the patients with the FTPHF, the main point to be noted is the reported complications of quite different rates. If an evaluation is carried out in terms of complications, higher complication rates have been found to be reported in the literature for patients whose index treatment is hemiarthroplasty.^6,11,12^ One explanation for this may be that it is possible to overcome problems such as cephalic collapse or osteonecrosis with the RTSA in patients whose index treatment is conservative or ORIF with lower complication rates. However, bone loss after removal of the implant is a problem that negatively affects the outcomes of the RTSA treatment in patients whose index treatment is hemiarthroplasty. In patients whose index treatment was hemiarthroplasty, Levy et al.^6^ reported complication rates as 28% (infection in one, humerus fracture in one, loosening in one, radial nerve palsy in one, dislocation in four of 29 patients), whereas Holschen et al.^12^ reported it as 32% (infection and instability in two, scapular fracture in three, symptomatic loosening in one of 35 patients). On the other hand, lower complication rates have been reported in the RTSA treatment of patients whose index treatment was conservative or ORIF.^10,17^ Willis et al.^17^ reported no complications in patients whose index treatment was conservative, whereas Santana et al.^10^ reported complication rates as 11% (infection in one of nine patients). In patients whose index treatment was ORIF, Santana et al. reported complication rates as 17% (dislocation in two and infection in one of 18 patients).^10^ Similarly, Gruphofer et al. reported complication rates as 19% (periprosthetic fractures in two, deep infection in two, fracture-dislocation in three, and glenoid loosening in three of 54 patients).^16^ The relatively low complication rates reported are noteworthy compared to the 27% complication rate (59 complications in 203 patients), which was previously reported in a study by Boileau et al., in which index treatment was conservative in 65% of the patients and ORIF and percutaneous pinning in 35%.^5^

No major complications was observed in any of the three different subsets with a mean follow-up of 37.85 (range: 24-83) months in our study. However, grade 1 scapular notching was detected in four patients and grade 2 scapular notching was detected in one patient. The authors believe that lower complication rates reported in this study probably resulted from increased surgical experience for the RTSA, better knowledge of possible complications, and taking precautions for these.

The present study has some limitations. The main limitations are its retrospective and two-center study design and relatively short follow-up duration [37.85 (range: 24-83) months]. Another limitation of the study is that different design prostheses (Depuy Delta Xtend, Biomet) were applied with different techniques (cemented/non-cemented) by two surgeons. Outcomes of the subsets according to the index treatment before the RTSA were not compared to each other because the numbers were too low to draw any substantial meaningful comparison. Comparing these subsets in a larger sample may be the subject of future studies. Nevertheless, we believe that this study contributes to the literature, given that the outcomes of treatment with the RTSA for the FTPHF were considerably favorable without any significant complication regardless of index treatment.

The findings of the present study support that treatment with the RTSA for the FTPHF seems to be a reliable treatment modality with favorable radiological and functional outcomes and acceptable complication rates.

**HIGHLIGHTS**

- In cases with failed treatment of proximal humeral fracture (FTPHF), patients can experience substantial pain and very limited functionality due to tuberosity malunion or nonunion, avascular necrosis, joint incongruity, and stiffness.
- Former studies reported poor outcomes and high complication rates with reverse total shoulder arthroplasty (RTSA) for the FTPHF.
- Our study demonstrated that favorable radiological and functional outcomes would be obtained by the RTSA for the FTPHF regardless of the initial treatment method probably owing to improved prosthetic designs and increased surgical experience for the RTSA.

## Figures and Tables

**Table 1.** Patient Demographics

Variable	
*n*	20
Age (years), mean (range)	71.35 (range: 54-81) years
Gender	17 F/M 3
Follow-up from the RTSA (months), mean (range)	37.85 (range: 24-83)
Duration from index treatment to RTSA (months)	21.25 (range: 1-86)

*n*, number; RTSA, reverse total shoulder arthroplasty; ORIF, open reduction and internal fixation; F, female; M, male.

**Table 2.** Treatment Details of Each Patient Treated with RSTA for Failed Tretmant of Proximal Humerus Fracture

Case No.	Sex/Age, year	Side	Dominant Side	Comorbidities	Index Treatment	Numbers of Surgeries	Time from Fracture to RSTA, months	Follow-up Time, months	Brand of RTSA	Cement	Approach	Subscap. Repair	Tubercle osteotomy	Compl.
1	F/71	R	YES	–	Conservative	0	5	25	*Depuy Delta Xtend*	-	Deltopectoral	+	–	–
2	F/83	L	NO	Hypothyroidism, HT	Conservative	0	83	26	*Depuy Delta Xtend*	+	Deltopectoral	+	–	–
3	F/66	R	YES	RA	Conservative	0	14	58	*Depuy Delta Xtend*	+	Deltopectoral	+	–	–
4	F/66	R	YES	–	Conservative	0	18	33	*Biomet*	+	Deltopectoral	–	–	–
5	F/87	R	YES	HT	Conservative	0	24	24	*Biomet*	-	Deltoid split	–	+	–
6	F/73	R		–	ORIF	1	18	31	*Depuy Delta Xtend*	-	Deltopectoral	+	–	–
7	F/66	R	YES	RA	ORIF	1	1	32	*Depuy Delta Xtend*	+	Deltopectoral	–	+	–
8	M/65	R	YES	–	ORIF	3	14	28	*Biomet*	-	Deltopectoral	–	no tubercle	–
9	M/80	L	NO	–	ORIF	3	34	60	*Depuy Delta Xtend*	+	Deltopectoral	+	–	–
10	F/73	L	NO	HT	ORIF	1	86	26	*Biomet*	-	Deltopectoral	–	–	–
11	F/74	L	NO	–	ORIF	1	5	54	*Biomet*	-	Deltopectoral	+	+	–
12	F/72	L	NO	DM type 2, Coron. Artery Dis.	ORIF	1	2	38	*Biomet*	-	Deltopectoral	+	–	–
13	M/71	R	YES	DM type 2	ORIF	1	3	24	*Biomet*	-	Deltopectoral	+	–	–
14	F/81	R	YES	DM type 2, Coron. Artery Dis.	ORIF	1	7	25	*Biomet*	-	Deltopectoral	+	no tubercle	–
15	F/81	R	YES	–	Hemiarthroplasty	1	12	83	*Depuy Delta Xtend*	+	Deltopectoral	+	–	–
16	F/80	L	NO	Hypothyroidism, HT	Hemiarthroplasty	1	16	48	*Depuy Delta Xtend*	+	Deltopectoral	–	+	–
17	F/65	R	YES	Parkinson's disease	Hemiarthroplasty	1	7	26	*Biomet*	+	Deltopectoral	–	–	–
18	F/62	R	YES	HT	Hemiarthroplasty	1	48	45	*Depuy Delta Xtend*	+	Deltopectoral	–	–	–
19	F/54	R	YES	Hypothyroidism	Hemiarthroplasty	1	15	44	*Biomet*	+	Deltopectoral	+	no tubercle	–
20	F/57	L	NO	–	Hemiarthroplasty	3	13	27	*Depuy Delta Xtend*	+	Deltopectoral	+	no tubercle	–
Mean or No.	17 F and 3 M/71.35 ± 4.15	13 R and 7 L	13 dominant and 7 non-dominant	3 Hypothyroidism, 5 HT, 2 Coron. Artery Dis., 3 DM type 2, 1 Parkinson's disease		1.05 ± 0.94	21.25 (interval 1-86 min)	37.85 (interval: 24-83)	10 *Depuy Delta Xtend*, 10 *Biomet*	11 cemented and 9 uncemented	19 Deltopectoral and 1 deltoid split	12 repair, 3 osteotomy, and 5 no repair		no compli-cation

No, number; RTSA, reverse total shoulder arthroplasty; Subscap,. subscapularis; Compl, complications; ORIF, open reduction and internal fixation; F, female; M, male; R, right; L, left; HT, hypertension; RA, rheumatoid arthritis; Coron. Artery Dis., coronary artery disease; DM, diabetus mellitus.

**Table 3.** Clinical Outcomes

Preoperative								Postoperative					
Case No.	Index Treatment	Anterior Flexion	Internal Rotation	External Rotation	Constant Score	ASES Score	VAS Score	Anterior Flexion	Internal Rotation	External Rotation	Constant Score	ASES Score	VAS Score
1	Conservative	45	Gluteal	10	35	35	5	120	L5	30	74	71.6	1
2	Conservative	90	Gluteal	0	27	28.3	8	160	T10	45	86	86.6	0
3	Conservative	60	Gluteal	0	29	21.6	7	110	L5	20	61	66.6	2
4	Conservative	20	L5	0	19	25	6	90	L3	10	57	56.6	3
5	Conservative	45	L5	20	21	25	6	90	L3	10	57	56.6	3
Mean or median		52 ± 25.6	Gluteal	6 ± 8.9	26.2 ± 6.4	27 ± 5.1	6.4 ± 1.1	114 ± 28.8	L3-L5	23 ± 14.9	67 ± 12.7	67.6 ± 12.5	1.8 ± 1.3
6	ORIF	40	Gluteal	20	24	21.6	7	90	L5	20	57	56.6	2
7	ORIF	60	Gluteal	20	31	21.6	7	80	L5	20	58	61.6	1
8	ORIF	0	Gluteal	0	17	11.6	8	95	L3	30	62	71	2
9	ORIF	20	Gluteal	10	17	15	8	120	L3	30	59	78	1
10	ORIF	60	L5	−20	39	43.3	3	100	Gluteal	20	6	70	3
11	ORIF	40	Trochanteric	10	16	11	7	90	L5	20	59	79	2
12	ORIF	20	Trochanteric	0	18	6	5	90	Gluteal	10	63	70	0
13	ORIF	70	L5	30	18	10	6	100	L5	30	60	77	1
14	ORIF	20	Trochanteric	10	12	13.3	8	90	Gluteal	20	65	78.3	2
Mean or median		36.7 ± 23.5	Gluteal	8.9 ± 14.5	21.3 ± 8.6	17 ± 11.1	6.6 ± 1.7	95 ± 11.2	L5	22.2 ±6.7	54.3 ± 18.3	71.3 ± 7.9	1.56 ± 0.9
15	Hemiarthroplasty	30	Gluteal	0	12	8.3	8	110	L5	20	63	59.9	3
16	Hemiarthroplasty	35	L5	20	19	10	6	120	L3	30	76	75	1
17	Hemiarthroplasty	10	Gluteal	0	12	8.3	7	135	L5	30	78	80	0
18	Hemiarthroplasty	30	Gluteal	20	25	25	6	110	Gluteal	20	61	65	2
19	Hemiarthroplasty	40	Gluteal	20	28	15	6	100	Gluteal	20	67	86	2
20	Hemiarthroplasty	10	L5	10	20	8	7	100	L5	20	66	78	3
Mean or median		25.9 ± 12.8	Gluteal	11.7 ± 9.8	19.3 ± 6.6	12.4 ± 6.7	6.7 ± 0.8	112.5 ± 13.3	L5	23.3 ± 5.2	68.5 ± 7	74 ± 9.8	1.83 ± 1.2

No, number; ASES, American Shoulder and Elbow Surgeons; VAS, visual analog scale; ORIF, open reduction and internal fixation.
